# Stationary Intraoral Tomosynthesis: A Step toward the Future of Routine Dental Imaging 

**DOI:** 10.30476/dentjods.2026.109821.2917

**Published:** 2026-06-01

**Authors:** Shahram Hamedani, Sepideh Zaeri

**Affiliations:** 1 Oral and Dental Disease Research Center, School of Dentistry, Shiraz University of Medical Sciences, Shiraz, Iran.; 2 Student Research Committee, Shiraz University of Medical Sciences, Shiraz, Iran.

**Keywords:** Carbon nanotube (CNT), Proximal overlap, 2D and 3D images, Bitewing technique, Vertical root fracture (VRF)

## Abstract

Stationary intraoral tomosynthesis (s-IOT) is an innovative imaging modality designed to overcome limitations of conventional bitewing radiography, notably proximal overlap. Based on carbon nanotube field emission technology, s-IOT acquires multiple projections across a 12° span and reconstructs them into high-resolution tomographic slices. Research indicates lower radiation dose compared to standard techniques and improved detection of proximal caries, periodontal bone loss, root fractures, and complex root morphologies including the direction and degree of dilaceration. Integration with synthetic radiography reduces artifacts and enhances interpretation. Future developments combining s-IOT with artificial intelligence and dual-energy imaging may further expand diagnostic potential, positioning s-IOT as a promising clinical tool.

**Letter to Editor**

Detecting proximal caries is often a challenging task in radiological diagnosis. Conventional imaging methods, particularly the bitewing technique may suffer from some technical and operational limitations. Overlapping the proximal areas in this modality is an issue of concern [ 1 ]. With advancements in dentomaxillofacial radiological techniques, stationary intraoral tomosynthesis (s-IOT) has been developed as an innovative technique that may revolutionize diagnostic approaches [ 2 ]. 

The concept of s-IOT was originally based on the tuned aperture computed tomography (TACT) technique [ 2 ]. TACT is an image reconstruction method that combines multiple 2D images to reconstruct a 3D image using an advanced algorithm [ 3 ]. However, TACT had several limitations, including the need for movement of the X-ray tube [ 2 ], longer imaging time [ 3 ] and reduced imaging resolution [ 3 ]. As a result, this technique has not yet been implemented clinically. To overcome these limitations and make the technology suitable for clinical use, the first prototype of the s-IOT system was designed at the University of North Carolina (UNC) in collaboration with the Department of Physics and Astronomy [ 2 ].

The purpose of this innovation was to address the limitations of conventional techniques. One of the fundamental changes was the replacement of the conventional cathode with a carbon nanotube (CNT). Compared to conventional techniques, s-IOT uses field emission to generate electrons [ 2 ]. Additionally, in the s-IOT system, several focal spots have been employed linearly with equal spacing [ 2 ]. These focal spots acquire multiple images parallel to the detector over a 12-degree angular span. Furthermore, a CMOS detector is fixed in a holder equipped with magnets at its end. These magnets ensure that the detector is positioned at a precise distance of 400 mm from X-ray sources [ 3 ]. 

Moreover, s-IOT is integrated with a proprietary image reconstruction algorithm. This algorithm acquires multiple 2D projections of the teeth from various angles and combines them mathematically to produce a 3D image. This enables the system to generate multiple image slices, each representing 0.5 mm of tooth thickness [ 3 ]. Beyond the similarity in exposure parameters, a comparative study was conducted to investigate the effective dose (E) of s-IOT and conventional techniques. The results show that the E dose for s-IOT with rectangular collimation is lower than that of radiography with circular collimation [ 4 ].

The results of another study illustrated that integrating synthetic radiography with s-IOT can reduce artifacts. This is especially true for artifacts adjacent to metallic restorations and obturation materials. This research also confirmed that synthetic radiography generates 2D images from 3D s-IOT datasets to enhance diagnostic efficiency, provide a format more familiar to clinicians, and improve interpretation [ 5 ].

In this regard, a study showed that s-IOT images have considerably less interproximal overlap (median 1%) compared to standard bitewing radiographs (median 13%). This improvement enables clinicians to scroll through the reconstructed slices and open the contact point between teeth [ 1 ]. In addition, image acquisition with s-IOT demonstrates higher contrast and identifies additional features. This enhanced contrast enables effective evaluation of periodontal bone loss, which is crucial for implant assessment. This modality also detects fractures by scrolling through reconstructed images in the buccal-to-lingual direction. This allows visualization of their depth and propagation. Since the s-IOT system acquires images of teeth from multiple angles, it has a higher likelihood of detecting fractures, especially those that may be missed by conventional techniques. Besides, s-IOT may provide accurate information about direction and degree of dilaceration by allowing visualization through multiple layers [ 3 ]. 

This modality may see greater adoption among endodontists due to its potential for diagnosing vertical root fractures (VRFs) and detecting complex root morphologies [ 5 ].

Despite its advantages, s-IOT has limitations, including potential sensitivity to patient movement during image acquisition and inherent limits in spatial resolution (similar to other conventional imaging modalities). Furthermore, reconstruction artifacts, particularly those adjacent to metallic restorations, remain a challenge. A critical consideration is that the technology has not yet been implemented clinically, as its development is currently restricted to a single research prototype at the UNC. Consequently, the number of clinical validation studies remains relatively small [ 3 ].

Moreover, combining s-IOT with synthetic radiography and artificial intelligence (AI) could significantly enhance both the efficiency and accuracy of dental imaging. Integrating AI could further simplify the diagnostic workflow. It may automatically detect and highlight pathological findings across the reconstructed dataset. As a result, the need for manual scrolling could be reduced and the risk of missed lesions may be minimized. Like conventional techniques, s-IOT cannot differentiate between soft and hard tissues. Combining the dual energy technique with s-IOT and AI might enable detection of periodontal pocket depth. This integrated approach may reduce the need for manual probing and create a data bank suitable for patient follow-up. 

In conclusion, s-IOT has great potential to improve diagnostic accuracy by reducing interproximal overlap and enabling assessment of bone loss, complex root morphology, and detection of vertical root fractures (VRFs) [ 3 ]. Nevertheless, further clinical studies are needed to confirm these findings. 
